# Choriocapillaris Changes Are Correlated With Disease Duration and MoCA Score in Early-Onset Dementia

**DOI:** 10.3389/fnagi.2021.656750

**Published:** 2021-04-13

**Authors:** Shuting Zhang, William Robert Kwapong, Tang Yang, Peng Liu, Qingzhang Tuo, Yajun Cheng, Xue Li, Ming Liu, Peng Lei, Bo Wu

**Affiliations:** ^1^Department of Neurology, West China Hospital, Sichuan University, Chengdu, China; ^2^Department of Emergency, West China Hospital, Sichuan University, Chengdu, China; ^3^State Key Laboratory of Biotherapy, West China Hospital, Sichuan University, Chengdu, China

**Keywords:** choriocapillaris flow density, Montreal cognitive assessment, duration, optical coherence tomography angiography, hippocampus

## Abstract

**Purpose**: Imaging of the choroid may detect the microvascular changes associated with early-onset dementia (EOD) and may represent an indicator for detection of the disease. We aimed to analyze the *in vivo* choriocapillaris (CC) flow density in EOD patients using optical coherence tomography angiography (OCTA) and evaluate the association with its clinical measures.

**Methods**: This cross-sectional study used the OCTA to image and analyze the choriocapillaris (CC) of 25 EOD patients and 20 healthy controls. Choriocapillaris flow density in the 3 mm area and 6 mm area was measured by an inbuilt algorithm in the OCT tool. Brain volume using magnetic resonance imaging and cognitive assessment was done and recorded.

**Results**: Significantly reduced capillary flow density of the choriocapillaris was seen in EOD patients when compared to healthy controls in the 3.0 mm (*P* = 0.001) and 6.0 mm (*P* < 0.001) area respectively. Montreal Cognitive Assessment (MoCA) scores in EOD patients positively correlated with choriocapillaris flow density in the 3 mm area (Rho = 0.466, *P* = 0.021). Disease duration of EOD patients also negatively correlated with choriocapillaris density in the 3 mm area (Rho = −0.497, *P* = 0.008).

**Discussion**: Our report suggests that choriocapillaris damage may be a potential indicator of early-onset dementia. Microvascular impairment may be involved in the early phase of dementia without aging playing a role in its impairment.

**Clinical Trial Registration**: www.ClinicalTrials.gov, ChiCTR2000041386.

## Introduction

With the major upsurge in the aging population, dementia has been a major public health concern worldwide (Livingston et al., 2017). However, the high prevalence of dementia in the elderly can overshadow the importance of its occurrence in younger patients. Early-onset dementia (EOD), which is defined as dementia before age of 65 years old and often associated with genetic factors, can provide critical biological mechanisms that may apply to late-onset dementia (LOD). For instance, the high prevalence of inherited dementias in younger patients has led to the identification of causative genes and subsequent molecular mechanism of direct relevance to the more common sporadic disease seen in older patients.

Moreover, EOD is a good model to study the factors interacting with both dementia and the aging process, such as the vascular factor, whose contribution is often questioned in the dementia pathogenesis since the vessels are also undergoing degeneration during the aging process. Recent reports (Zlokovic, 2011; Laing et al., 2020) have shown that microvascular influences affecting cerebral microcirculation may contribute to the pathogenesis of dementia. Reports have also suggested that decreased cerebral blood flow occurs before the onset of clinical dementia which was trailed by decreased amyloid-beta clearance resulting in neurotoxicity (Kalaria et al., 2012; Cunha et al., 2017) making the possibility of vascular factor as a pre-clinical dementia biomarker.

Visual dysfunction such as loss of vision has been reported to be one of the earliest manifestations in dementia (Sadun et al., 1987; Katz and Rimmer, 1989) and some have suggested that these changes occur before the onset of dementia (Pillai and Cummings, 2013; Tzekov and Mullan, 2014). Reports have shown that these visual abnormalities are associated with the degeneration of the brain’s visual pathway (Javaid et al., 2016). Besides, recent reports have suggested that deposition of amyloid in the brain also occurs in the optic nerve (second cranial nerve) and retina which lead to neuroaxonal loss (Koronyo-Hamaoui et al., 2011; Javaid et al., 2016). With the association between the retina and the brain, *in vivo* reports have shown the structural changes occur in the retinal choroid during the pathogenesis of dementia (Bulut et al., 2016; Cunha et al., 2017). Sequentially, the retinal choroid has been proposed as a potential early and noninvasive microvascular indicator in the eye for neurodegeneration in the brain because of the abundance of microvasculature (Trebbastoni et al., 2016; Cunha and Castanheira-Dinis, 2017). Nonetheless, the clinical use of the retinal choroid as a reliable indicator for dementia remains questionable given varying findings. Previous reports focused predominantly on the total choroidal thickness with little attention on the choroidal microvasculature (choriocapillaris, CC).

Optical coherence tomography angiography (OCTA) allows the noninvasive *in vivo* visualization of the multiple capillary plexus in the retina and choroid. Previous reports have shown an enlargement of the foveal avascular zone (FAZ) and significant macula density loss in dementia patients; however, very little is known of the choroidal microvasculature in dementia patients. We hypothesize that the CC flow change might be correlated with the severity of EOD. In a cohort study of patients with EOD, we aimed to assess the *in vivo* choriocapillaris flow density (CFD) changes in early-onset dementia (EOD) patients and healthy controls using OCTA.

## Materials and Methods

### Study Design and Participants

In this study, we enrolled 28 EOD patients from the neurology department of West China Hospital. Twenty healthy controls were recruited as well. Demographic data such as body mass index, education level, age, and gender were recorded. All participants enrolled in this study underwent a Mini-Mental State Examination (MMSE) and Montreal Cognitive Assessment (MoCA). Clinical information of all participants was recorded. All EOD patients met the diagnostic criteria of EOD (Rossor et al., 2010) and the national institute of neurological speech disorders and stroke (NINCDS-ADRDA; McKhann et al., 1984). Subjects with histories of ocular or neurologic diseases that could affect the results such as high refractive errors of more than +5.0 or −6.0 diopters, age macular degeneration, diabetic retinopathy, glaucoma, cataracts, corneal diseases, cystic macular, coagulopathy, and uncontrolled hypertension were excluded. Other exclusion criteria were as follows: a history of symptomatic stroke or carotid stenosis of ≥50%, and other neurological disorders; a history of brain trauma, tumor. Intracranial infection and systemic inflammatory disease; contraindication for MRI; alcohol or drug abuse, and psychiatric disorders; local eye disorders that could cause optic fundus disease such as various eye inflammatory responses or eye surgeries (e.g., cataract extraction or laser surgery, severe cataract, glaucoma) within 6 months before enrollment. Healthy controls also followed the aforementioned exclusion criteria; participants with uncontrolled hypertension were also excluded. The study was approved by the Biomedical Research Ethics Committee and the Committee on Human Research of West China Hospital, Sichuan University (2020-104). Informed consent was obtained from participants or their guardians.

### Three-Dimensional Magnetic Resonance Imaging (MRI) Scanning and Brain Volume Measurements

MRI scanning was performed on a 3-T MRI unit (Signa 750 W GE Healthcare, Milwaukee, WI, USA). The scanning protocol was as follows: (i) whole brain 3 D-T1 BRAVO sequence (TR/TE 8.5/3.2 ms; Prep time: 450 ms, flip angle 12°; voxel size 1.0 × 1.0 × 1.0 mm); (ii) T2 FLAIR (TR/TE/TI 9,000/95/2,474 ms; voxel size 0.93 × 0.93 × 5.0 mm; gap 1 mm); (iii) T2 propeller (TR/TE 5,039/110 ms; voxel size 0.58 × 0.58 × 5.0 mm; gap 1 mm); and (iv) 3D-ASL (TR/TE 4,809/10.7 ms; slice thickness 4 mm; Post label delay 2,024 ms; arms 8; number of excitation 3) during resting state, subjects were told not to concentrate on any particular subject, but just to relax with their eyes closed. The complete scanning protocol took 20 min. Brain structure volume was automatically measured by AccuBrain™ brain structure volumetry tool (Abrigo et al., 2019) as shown in Table 2 (Supplementary Figure 1).

**Table 1:** Baseline characteristics of the study population.

	EOD	HC	*P*-value
Number	25	20	
Number of eyes	41	39	
Gender	15/10	11/9	
Age (years)	61.04 (5.45)	58.14 (2.98)	0.063
Education			
Illiteracy	3	2	
Elementary school	5	4	
Middle school and higher	17	14	
Duration (years)	2.4 (1.56)		
MMSE score	12.62 (5.04)	28.29 (1.33)	<0.001
MoCA score	11.15 (8.06)	26.71 (1.33)	<0.001
Ophthalmology exam
IOP (mmHg)	13.84 (2.01)	14.01 (1.98)	0.862
VA (Snellen chart)	0.70 (0.27)	1.04 (0.11)	<0.001
VA (LogMAR)	0.16 (0.16)	-0.18 (0.05)	<0.001

*Descriptive statistics were calculated using mean (SD) for continuous variables. Abbreviations: AD, Alzheimer’s disease; HC, healthy controls; MMSE, Mini-Mental State Examination; MoCA, Montreal Cognitive Assessment; IOP, intraocular pressure; VA, visual acuity; LogMAR, logarithm of the minimum angle of resolution*.

**Table 2:** Magnetic resonance imaging variables of EOD patients.

MRI variables	Mean (SD)
**Volume (ml)**
Total volume with CSF	1,373.84 (124.97)
Total volume	1,027.59 (99.21)
Hippocampus	5.63 (1.05)
Left hippocampus	2.78 (0.51)
Right hippocampus	2.85 (0.57)
**Ratio**
Total volume with CSF	98.72 (6.4)
Total volume	72.47 (11.68)
Hippocampus	0.43 (0.13)
Left hippocampus	0.23 (0.16)
Right hippocampus	0.24 (0.16)

*MRI, magnetic resonance imaging; CSF, cerebrospinal fluid*.

### Ophthalmic Examination

Enrolled participants underwent comprehensive ophthalmic examination including intraocular pressure (IOP), visual acuity under illumination, fundus imaging using the fundus camera, and spectral-domain optical coherence tomography examination.

### Acquisition of OCTA Images

**Figure 1:**
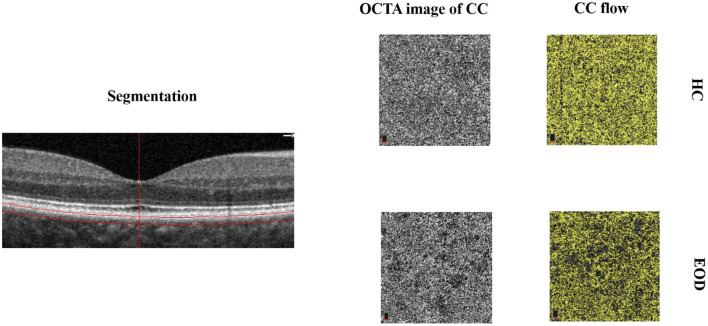
Segmentation and representative image of the choriocapillaris (CC). The choriocapillaris is the microvessels within the Bruch’s membrane and the upper boundary of the stroma. Early-onset dementia (EOD) showed interrupted choriocapillaris in the optical coherence tomography angiography (OCTA) image. The CC flow showed reduced choriocapillaris density when compared with healthy controls.

The RTVue XR Avanti Spectral Domain OCT system (Optovue, Inc., Fremont, CA, USA) equipped with AngioVue software was used to image each participant’s eye. While centered on the fovea, Angio Retina 3.0 mm and HD Angio Retina 6.0 mm were imaged in each participant. The choroidal microvessels, choriocapillaris, were defined as the microvessels within the Bruch’s membrane and the upper boundary of the stroma (Figure 1). Motion correction and 3D projection artifact reduction were done by the Avanti system of the OCTA tool. The choriocapillaris (CC) was evaluated by the in-built software of the OCTA tool as previously detailed (Yang et al., 2019). The capillary density in the CC was defined as the percentage (%) occupied by the microvasculature in the analyzed area i.e., 3 × 3 mm and 6 × 6 mm. Images with signal quality (SQ) less than 6 were excluded (Lim et al., 2018). Images with motion artifacts seen on the *en face* images or irregular/blurred segmentation of the choriocapillaris were also excluded from our data analyses. Images included in our data analyses were of good quality.

### Statistical Analyses

SPSS software (version 22) was used to perform statistical analyses. Data were expressed as the mean ± standard deviation (SD). A generalized estimating equation (GEE) was used to compare the differences between the choriocapillaris flow density (both 3 mm^2^ and 6 mm^2^) between the healthy controls and the EOD group while adjusting for inter-eye dependencies, SQ, and risk factors (hypertension, diabetes, age, and gender, and educational level). Pearson correlation was used to evaluate the association between the OCT parameters, MRI variables, and clinical variables in EOD participants. *P*-values less than 0.05 (*P* < 0.05) were considered to be statistically significant.

## Results

A total of 28 EOD patients were screened and three EOD patients were excluded because of incompletion of OCTA examination. Twenty-five EOD patients (mean age 61.0 ± 5.5 years) and 20 healthy controls (HC, mean age 58.1 ± 3.0 years) were included in the final analyses (Table 1). The mean duration of EOD patients was 2.4 ± 1.56 years; their mean cognitive assessment scores were as follows: MMSE score =12.62 ± 5.04 and MoCA score = 11.15 ± 8.06 as shown in Table 1.

Forty-one eyes from 25 EOD patients and 39 eyes from 20 healthy controls were included in our data analyses. Ten eyes were excluded owing to poor image quality (SQ < 6) and motion artifacts. EOD patients (7.70 ± 1.09) had significantly lower (*P* < 0.001) SQ when compared with healthy controls (8.90 ± 0.88). Demographics, clinical variables, and MRI parameters are shown in Table 1. Figure 1 shows the en face OCTA image of the choriocapillaris and the angiograms of the choriocapillaris flow area (yellow) in EOD and healthy controls. Interrupted choriocapillaris was seen in the en face OCTA image of EOD patients while showing reduced choriocapillaris flow area when compared with healthy controls (Figure 1).

### Comparison of OCTA Parameters Between EOD and Healthy Controls

EOD patients (63.79 ± 4.16%) showed significantly reduced choriocapillaris density (*P* = 0.001, Table 3) in the 3 × 3 mm area when compared with healthy controls (67.20 ± 3.08%). In the 6 × 6 mm area, EOD patients (66.35 ± 3.15%) also showed significantly reduced choriocapillaris density (*P* < 0.001, Table 3) when compared with healthy controls (70.85 ± 1.85%).

**Table 3:** Comparison of the choriocapillaris flow density.

	EOD	HC	*P*-value
Choriocapillaris 3 mm, %	63.79 (4.16)	67.20 (3.08)	0.001
Choriocapillaris 6 mm, %	66.35 (3.15)	70.85 (1.86)	<0.001

*Data were adjusted for inter-eye dependencies, SQ, risk factors (hypertension, diabetes, age, and gender), and educational level. Abbreviations: EOD, early-onset dementia; HC, healthy control; SQ, signal quality*.

### Association Between Choriocapillaris Densities and Clinical Variables

MoCA scores in EOD patients positively correlated with choriocapillaris flow density in the 3 mm area (Rho = 0.466, *P* = 0.021; Table 4) but did not significantly correlate with choriocapillaris flow in the 6 mm area (Rho = 0.321, *P* = 0.118; Table 4). Visual acuity (LogMAR) in EOD patients also significantly correlated with the choriocapillaris flow density in the 6 mm area (Rho = −0.222, *P* = 0.001). Disease duration of EOD patients also negatively correlated with choriocapillaris density in the 3 mm area (Rho = −0.497, *P* = 0.008; Table 4) but not with the choriocapillaris of the 6 mm area (Rho = −0.301, *P* = 0.094; Table 4).

**Table 4:** Correlation between choriocapillaris densities, MRI variables and clinical variables.

	MoCA	*P*-value
CC%, 3 × 3 mm	0.466	0.021
CC%, 6 × 6 mm	0.321	0.118
	Duration	*P*-value
CC%, 3 × 3 mm	−0.497	0.008
CC%, 6 × 6 mm	−0.301	0.094
Total hippocampus ratio	0.489	0.039
Ratio hippocampus volume (ml)	0.519	0.019

*CC, choriocapillaris; MoCA, Montreal cognitive assessment*.

### Association Between MRI Variables and MoCA Scores

MoCA scores showed significant correlation with the ratio of total hippocampus (Rho = 0.489, *P* = 0.039; Table 4) and right hippocampus volume (Rho = 0.519, *P* = 0.019; Table 4) respectively in EOD patients.

## Discussion

Our current study assessed the *in vivo* choriocapillaris density in EOD patients and the association with its clinical variables. Compared with healthy controls, EOD patients had significantly reduced choriocapillaris flow density. Moreover, the choriocapillaris flow density was negatively correlated with the disease duration and severity of cognitive impairment using MoCA respectively. Our data add to the notion that there is a significant difference in the choriocapillaris changes between EOD and healthy controls. Taken together with the structural choroidal changes that occur in dementia patients, our study suggests that choriocapillaris changes assessed with the OCTA may be potentially useful in monitoring the course of the disease.

The choroid, which lies between the retina and outer layer of the eye, accounts for the majority of blood supply to the retina. It is responsible for the blood supply to the outer retina which includes the retinal pigment epithelium (RPE) and photoreceptors, and some portions of the inner retina (McLeod and Lutty, 1994). OCTA has been reported to be a convenient and invasive method to screen and monitor dementia development (Zhang et al., 2020). Our current study used an in-built software in the OCTA tool to assess the choriocapillaris blood flow in EOD patients which helps the reduction of projection artifacts. The choriocapillaris flow density data and motion artifacts were corrected with the in-built algorithm which made our measurements more reliable as previously reported (Zhang et al., 2016). Our study showed that EOD patients had significantly reduced choriocapillaris density when compared with healthy controls suggesting that microvascular changes in the choriocapillaris could be a useful indicator for understanding the pathological mechanism assessing the disease. Our study expands the understanding of the microvascular changes which occur in the eye of EOD and how these changes could be useful indicators for assessing the disease.

The cerebrovascular mechanism of dementia was proposed for decades (Raz et al., 2016). The most controversial issue of vascular mechanism in dementia is the role of aging in vascular impairment; it has been shown that aging has a great effect on microvessels thus lowering their importance in the pathogenesis of dementia. Aging, which causes the atrophy of vessels, creates an ischemic condition that affects the choroid, the powerhouse of blood supply in the eye as previously reported (Wildsmith et al., 2013). The significant reduction in the choriocapillaris flow area after adjusting for age shows that EOD primarily affects the choriocapillaris without the effect of the aging process. With the choroidal vessels being the powerhouse of the posterior segment, our report suggests that the choriocapillaris is significantly affected in EOD. As such, the significant reduction in the flow density of the choriocapillaris of our EOD patients may be due to the distinctive microvascular pathology associated with dementia itself.

Fundamental methods for diagnosis of AD are based on neuropsychological assessment such as Mini-Mental State Examination scores (MMSE; Pasi et al., 2015) and Montreal Cognitive Assessment (MoCA; Horton et al., 2015), which are used to evaluate the cognitive status in dementia patients. The association between OCT parameters and these neuropsychological assessments can be useful in the clinical evaluation and monitoring of patients with dementia, as the severity of cognitive impairment can be measured. A novel finding in our current study was an association shown between the choriocapillaris flow density and the MoCA scores. The association between MoCA scores and choriocapillaris may suggest that MoCA is sensitive to microvascular damage as previously reported (Pasi et al., 2015). Our present study also showed that disease duration was associated with reduced choriocapillaris flow density; the negative association suggests that the longer the duration of dementia, the greater the reduction in choriocapillaris flow density in EOD patients and vice versa.

Besides, our current study found a significant correlation between reduced visual acuity and choriocapillaris flow density in EOD patients indicating that dysfunction of vision is associated with reduced choriocapillaris flow density. Visual dysfunction such as loss of visual acuity has been reported to be one of the earliest clinical manifestations in dementia (Sadun et al., 1987). Instances where retinal homeostasis is impaired by the development and progression of a disease, the outer retina (mainly the photoreceptors, which is responsible for the acuity of vision and receives its oxygenation from the choriocapillaris) may be highly susceptible to modifications in the microcirculation of the choroid (Soukup et al., 2019). As such, we speculate that reduced choriocapillaris flow density in EOD contributes to dysfunction of vision in the disease cascade of dementia.

Hippocampal atrophy, a marker which can be assessed with MRI, is often used as an indicator for neurodegeneration in dementia. Accumulating MRI studies (Du et al., 2001; van de Pol et al., 2007) have shown significant reduction of hippocampal volume in dementia patients when compared with healthy controls. Our current study found positive correlation between MoCA score and total hippocampal ratio in EOD patients; we also showed that smaller right hippocampal volume was associated with MoCA scores in EOD patients. The degeneration of hippocampal volume is often associated with cognitive impairment where both positive and negative relationships have been found (Van Petten et al., 2004). However, the positive correlation seen in our report suggests that reduced MoCA scores reflects the hippocampal structure in EOD patients and vice versa.

The major limitation of the study is the observational cross-sectional design. Another limitation is our small sample size. Thus, longitudinal studies with larger sample sizes are needed to validate our speculations. Our study did not evaluate the thickness of the choroid, deeper vessels of the choroid, and outer retina; we used the in-built software of the OCTA tool which provides the microvascular density of the choriocapillaris. The wavelength of the OCTA is not long enough to penetrate deeper into the choroid to image the deeper vessels of the choroid. Although previous reports have shown choroidal thinning in dementia/AD, these reports used different OCT machines or external software to segment and give a report on the choroid. Our current study relied on the in-built software of the OCTA machine which could give data on the choriocapillaris, the superficial microvasculature of the choroid, which is found beneath the Bruch membrane; therein, future studies are needed to evaluate the choroidal and outer retinal thickness with the microvasculature of the choroid. Our current report did not assess the microvasculature of the brain and is another limitation in our study. *In vivo* imaging of the cerebral microvasculature is extremely challenging due to the extremely scattering properties of the skull and the relatively high optical density of the brain cortex. Besides, the size of the cerebral microvasculature is small (3—7 μm in diameter) and *in vivo* imaging may be challenging due to the resolution of the MRI tool. The inclusion of dementia patients with a different gene mutation may be another limitation in our study.

In conclusion, our study showed that EOD patients have significantly reduced choriocapillaris flow density. Our report suggests that choriocapillaris damage may be a potential indicator of early-onset dementia and its impairment may be involved in the earliest phase of dementia without aging playing a role in it. We also showed that microvascular damage in the choriocapillaris is associated with cognitive impairment using MoCA. Our study suggests that imaging of the choriocapillaris may help in monitoring the disease progression and facilitate the evaluation of some therapies for dementia.

## Data Availability Statement

The raw data supporting the conclusions of this article will be made available by the authors, without undue reservation.

## Ethics Statement

The studies involving human participants were reviewed and approved by West China Hospital of Sichuan University. The patients/participants provided their written informed consent to participate in this study.

## Author Contributions

SZ, BW, WK, ML, and PLe designed and conducted the cohort study. SZ, WK, TY, PLi, YC, and XL collected the data and constructed the database. SZ, WK, TY, PLi, and QT analyzed the data. SZ, BW, and WK wrote the article. All authors contributed to the article and approved the submitted version.

## Conflict of Interest

The authors declare that the research was conducted in the absence of any commercial or financial relationships that could be construed as a potential conflict of interest.

## Supplementary Material

SUPPLEMENTARY FIGURE 1 Sample of total brain volume and hippocampus volume measurements by AccuBrain™.
