# Supplementary figures and images for: Choriocapillaris Changes Are Correlated With Disease Duration and MoCA Score in Early-Onset Dementia
Source: Front Aging Neurosci. 2021 Apr 13;13:656750. doi: 10.3389/fnagi.2021.656750 (PMC8076507; doi:10.3389/fnagi.2021.656750)

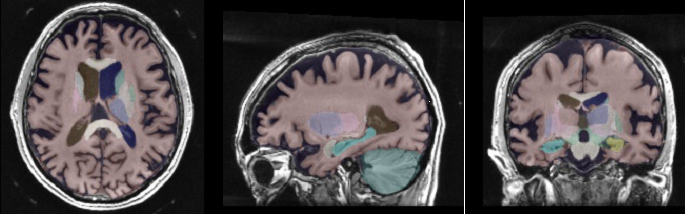

Supplement: SUPPLEMENTARY FIGURE 1 — Sample of total brain volume and hippocampus volume measurements by AccuBrain™. [file Image_1.TIF]
